# Screening and Characterization of Antiglycoxidant Anthocyanins from *Vaccinium myrtillus* Fruit Using DPPH and Methylglyoxal Pre-Column HPLC Assays

**DOI:** 10.3390/antiox9060512

**Published:** 2020-06-10

**Authors:** Didier Fraisse, Alexis Bred, Catherine Felgines, François Senejoux

**Affiliations:** Human Nutition Unit and ECREIN Team, Université Clermont Auvergne, INRA, UNH, F-63000 Clermont-Ferrand, France; didier.fraisse@uca.fr (D.F.); alexis.bred@uca.fr (A.B.); catherine.felgines@uca.fr (C.F.)

**Keywords:** anthocyanins, delphinidin, petunidin, cyanidin glycosides, bilberry, pre-column HPLC method, antioxidant, glycation, BSA/D-ribose assay

## Abstract

*Vaccinium myrtillus* fruit (bilberry) is well known for its high richness in anthocyanins, which may be responsible for its preventive effects on several oxidative and carbonyl stress-related pathologies. However, limited data are available regarding the antioxidant and antiglycative contributions of its constituents. Spectrometric analyses were performed to evaluate anthocyanin content, radical scavenging and antiglycative properties of an anthocyanin-rich extract from bilberries. Additionally, original DPPH and methylglyoxal pre-column HPLC methods were instigated to allow straightforward identification of the main contributors to radical and carbonyl trapping effects. Finally, representative pure anthocyanins were evaluated using classical DPPH and antiglycation assays. Delphinidin, petunidin and cyanidin glycosides were identified as the most effective radical scavenging constituents in both HPLC and spectrometric DPPH evaluations. Potent antiglycative activities were also assessed for cyanidin, delphinidin and petunidin glucosides as attested by their respective IC_50_ values of 114.2 ± 7.8, 130.5 ± 2.8, and 132.4 ± 3.7 µM. Interestingly, methylglyoxal spiking evaluation demonstrated that all bilberry anthocyanins exerted noticeable and comparable α-dicarbonyl trapping effects. Anthocyanins can be regarded as potent antiglycoxidant agents that might account for some health benefits of bilberries consumption. Besides, significant differences in their contributions were successfully highlighted by the employed pre-column HPLC assays.

## 1. Introduction

*Vaccinium myrtillus* L. (bilberry) is a low-branched shrub that belongs to the Ericaceae family. Also known as the European blueberry, this species produces dark blue edible fruits that can be eaten raw or cooked. A large variety of phenolic constituents has been described in *V. myrtillus* berries. Indeed, numerous flavonols including quercetin, myricetin, and isorhamnetin derivatives as well as tannins and phenolic acids have been reported [1,2]. Additionally, these fruits are considered as one of the most important sources of anthocyanins, with contents ranging from 100 mg to 500 mg per 100 g of fresh weight [3]. Of interest, it has been demonstrated that such constituents exert a wide range of biological activities including potent radical scavenging and anti-inflammatory effects [4,5]. More recently, several anthocyanin-rich extracts have been reported to inhibit in vitro formation of advanced glycation end products (AGEs) [6,7]. Of interest, the antiglycative activity of bilberries was further suggested by a clinical study reporting that human plasma protein-bound AGEs can be partly reduced by *V. myrtillus* extract supplementation [8]. All these reported activities might explain, at least in part, the positive effects of anthocyanin intake on human health. Indeed, several epidemiological or clinical data have highlighted the preventive effects of anthocyanin-rich food on oxidative stress and chronic pathologies such as cardiovascular diseases and diabetes [9,10].

The antiglycative and antioxidant properties of anthocyanins are assumed to play an important role in their benefits on human health. However, the activities of such constituents can strongly differ depending on their structures [11]. Hence, a basic estimation of total anthocyanin content from plants, fruits, or extracts might not efficiently reflect their biological effects. An important issue in the process of standardization of anthocyanin-containing products requires the characterization of most active constituents to provide adequate markers for quality control. Of interest, such a procedure is of particular importance concerning *V. myrtillus* in view of the numerous reported cases of adulteration of marketed products [12]. The conventional bio-guided fractionation of plant extracts is a long-lasting, labor-intensive, and costly task. Consequently, to hasten the identification of bioactive compounds, original and effective methods have been developed to allow straightforward detection of potential antioxidant and antiglycative constituents from complex plant matrices. For instance, HPLC analysis coupled with pre-column DPPH assay has been successfully applied in several phytochemical investigations [13]. This method consists of spiking extracts with a DPPH^•^ solution prior to chromatographic evaluation and relies on the ability of antioxidant constituents to be transformed upon reaction with radical species. On this basis, the peak area of bioactive compounds is diminished in the chromatogram of spiked extracts, whereas inactive constituents are not affected by any modifications. This kind of procedure has been similarly applied to other radical species including ABTS^•+^ [14], nitric oxide [15], and peroxynitrite scavengers [16]. In the same way, HPLC spiking experiments have also been designed to seek out natural products capable of reacting with α-dicarbonyl compounds such as methylglyoxal (MGO) or glyoxal [17]. Generated through different pathways including non-enzymatic degradation of reducing sugars, these highly reactive carbonyl species are indeed reported to be key intermediates in the formation of AGEs [18].

The present study was thus aimed at performing a qualitative and quantitative chemical investigation of an anthocyanin-rich extract from *V. myrtillus* (AEVM) by HPLC analyses. Additionally, spectrophotometric and fluorimetric evaluations were achieved to estimate its total anthocyanin content and radical scavenging properties as well as its inhibition effects on the formation of AGEs. Finally, a different set of HPLC spiking experiments were performed in order to directly identify the main contributors to the antiglycoxidant (i.e., antioxidant and antiglycative) activities of the studied extract.

## 2. Materials and Methods

### 2.1. Plant Material and Reagents

Bilberry anthocyanin-rich dry extract, Anthocyan^®^, was supplied by Ferlux SA (Cournon d’Auvergne, France). Methanol was of analytical grade and purchased from Carlo Erba Reagents SAS (Val-de-Reuil, France). Acetonitrile (MeCN) was of chromatographic grade (Carlo Erba Reagents SAS, Val-de-Reuil, France). All aqueous solutions were prepared with pure water produced by Milli-Q water (18.2 MΩ) system (Merck, Darmstadt, Germany). Phosphoric acid (purity 85%) was purchased from VWR Prolabo (Fontenay-sous-Bois, France). Delphinidin 3-*O*-glucoside chloride, cyanidin 3-*O*-glucoside chloride, cyanidin 3-*O*-galactoside chloride, petunidin 3-*O*-glucoside chloride, peonidin 3-*O*-glucoside chloride, malvidin 3-*O*-glucoside chloride, and malvidin 3-*O*-galactoside chloride were purchased from Extrasynthese (Genay, France). Aminoguanidine chloride, MGO (40% in water), bovine serum albumin (BSA), D-ribose, 6-hydroxy-2,5,7,8-tetramethylchromane-2-carboxylic acid (Trolox) and DPPH were bought from Sigma–Aldrich Chemical (Saint-Quentin Fallavier, France). MGO and DPPH radical solutions were freshly prepared every day and kept protected from light.

### 2.2. Spectrometric Evaluations of Total Anthocyanin Content, Radical Scavenging, and Antiglycation Activities

Total anthocyanin content was estimated using the colorimetric method following European Pharmacopoeia procedure [19]. The amount of total anthocyanins was expressed as milligram of cyanidin 3-*O*-glucoside equivalent per gram of dry extract.

DPPH radical scavenging activity was spectrophotometrically assessed according to a previously published method, with slight modifications [20]. Briefly, sample solutions (pure compounds or extract) were extemporaneously prepared at concentrations between 0.05 mg/mL and 1 mg/mL in water, and 20 μL of each were mixed to 2.5 mL of fresh DPPH solution (25 μg/mL in methanol). After 30 min of incubation at room temperature, absorbance was measured at 515 nm using a UV–vis Jasco V-630 spectrophotometer (Lisses, France). A standard curve of Trolox in the range of 0.1–3 mM was constructed (R^2^ = 0.9978, y = 2752.8 x + 1.5196). Radical scavenging activities were respectively expressed in term of micromoles of Trolox equivalent (μmol TE) per gram of dry extract for AEVM and as IC_50_ for pure compounds.

Inhibition of AGEs formation was determined using BSA/D-ribose assay as previously reported by Derbré et al. [21]. Briefly, 40 μL of D-ribose (120 mM), 40 μL BSA (25 mg/mL), and 20 µL of the extract or pure compounds at different concentrations were incubated at 37 °C in a phosphate buffer, 50 mM, pH 7.4. After an incubation of 24 h, AGEs fluorescence was evaluated with a TECAN infinite F200 PRO microplate reader (Lyon, France) using 370 nm and 440 nm as the excitation and emission wavelengths. Aminoguanidine chloride was chosen as positive control, and results were indicated as IC_50_ values in µg/mL for extract and in µM for pure compounds.

### 2.3. HPLC Analysis of AEVM

HPLC analysis of the studied extract was performed using a LaChrom Elite system (VWR-Hitachi, Radnor, PA, USA) equipped with two L7100 pumps, a L7200 autosampler, a L2450 diode array detector (DAD) performing the wavelength scanning from 200 nm to 600 nm, and EZ Chrom Elite software (Agilent Technologies, Massy, France). AEVM (1 mg/mL) was chromatographed with a reversed phase Interchim^®^ UP3ODB C_18_ column (150 × 4.6 mm, 5 μm particle size). Gradient elution was developed with a mobile phase composed of water containing 1% phosphoric acid (A) and MeCN (B). The gradient was set as follows: 0–10 min, 9% B; 10–25 min, 9–12% B; 25–40 min, 12–16% B; 40–45 min, 16% B; 45–50 min, 16–40% B; 50–55 min, 9% B. A flow rate of 1 mL/min and an injection volume of 20 μL were selected. The quantification of individual anthocyanins was achieved by constructing calibration curves with commercial anthocyanin constituents that were representative of the five different groups occurring in bilberry (delphinidin, cyanidin, petunidin, malvidin and peonidin 3-*O*-glucosides). Standard solutions in the concentration range of 5–100 μg/mL were injected, and the chromatograms were monitored at a wavelength of 530 nm. Five-point calibration curves were constructed, and all of them exhibited satisfying linearities (R^2^ > 0.99). Results were expressed as mg of constituent per g of dry extract.

### 2.4. Pre-column DPPH-HPLC Evaluation of AEVM

DPPH-HPLC experiments were conducted as previously described by Meda et al. [22] with minor modifications of extract and DPPH concentrations. Briefly, solution of AEVM (1.5 mg/mL) were spiked with DPPH solutions of different concentrations (0.75 mM and 1.5 mM in MeOH) at the ratio of 2:1 (v/v). The mixtures were then vortexed and incubated for 30 min at room temperature prior to HPLC analysis using the above-described conditions. The control consisted of a solution of AEVM (1.5 mg/mL) mixed with MeOH at a ratio of 2:1 (v/v) and then similarly treated than DPPH spiked solutions. The percentage of remaining constituents was calculated as the ratio between the constituent peak area after reaction with DPPH divided by the constituent peak area in the control sample.

### 2.5. Pre-column MGO-HPLC Evaluation of AEVM

MGO-HPLC experiments were performed according to a previously published method [17] with slight modifications. Extemporaneously prepared solutions of AEVM (2 mg/mL in 50 mM phosphate buffer, pH = 7.4) were mingled with MGO solution (0.5 mM in phosphate buffer) at the ratio of 1:1 (v/v). The solutions were then vortexed and incubated for 1 h at 37 °C prior to HPLC analysis using the above reported method. The control contained a mixture of AEVM (2 mg/mL in phosphate buffer) and phosphate buffer at the ratio of 1:1 (v/v), which was prepared and incubated in the same way as the MGO spiked solutions. The percentage of remaining constituents was calculated as the ratio between the constituent peak area after reaction with MGO compared to the constituent peak area in control sample.

### 2.6. Statistical Analyses

The statistical significance of difference was analyzed by one-way ANOVA followed by Fisher’s Least Significant Difference (LSD) test and *p* values of 0.05 or less (*p* ≤ 0.05) were considered statistically significant. All data are indicated as the mean ± standard error of the mean (SEM). The AGEs assays were performed in quadruplicate, and all other spectroscopic and chromatographic analyses were done in triplicate.

## 3. Results and Discussion

### 3.1. Total Anthocyanin Content, DPPH Scavenging, and Antiglycative Activities of AEVM

Considering that anthocyanin constituents are regarded as major contributors to the antioxidant activity and health benefits of bilberries [8], an enriched extract was employed for the present investigation. Spectrometric estimation of its total anthocyanin content revealed a very high amount with an estimated value of 392.7 ± 7.7 mg of cyanidin 3-*O*-glucoside equivalents per gram of dry extract. The antioxidant properties of AEVM were further confirmed by performing a DPPH colorimetric assay. Indeed, the studied extract exerted very potent DPPH radical scavenging properties as ascertained by its activity of 3438 ± 129 µM of TE/g of dry extract. Such results are consistent with previous reports highlighting the powerful antioxidant effect of bilberry extracts using DPPH assay and other antioxidant models such as Oxygen Radical Absorbance Capacity (ORAC) or Ferric Reducing Antioxidant Power (FRAP) evaluations [23,24]. By contrast, very limited data are available regarding antiglycative properties of *V. myrtillus* fruits. By using a BSA/D-ribose assay, our experiments demonstrated that AEVM induced a potent inhibitory effect on AGEs formation. With an IC_50_ value of 71.69 ± 1.47 µg/mL, this extract exerted a significantly higher (*p* < 0.05) antiglycation effect than the positive control aminoguanidine chloride (264.7 ± 13.8 µg/mL). To the best of our knowledge, this is the first report of such activity for bilberries. This result can be tied in with previously published data on the potent antiglycation properties of fruit extracts from other *Vaccinium* species, such as *V. cylindraceum*, *V. padifolium*, and *V. angustifolium* [25,26]. Taken together, these preliminary results confirmed that AEVM exerted potent antioxidant and antiglycation properties, which might be attributable to its high anthocyanin content. HPLC experiments were thus implemented to determine the implication of each anthocyanin derivatives to the observed activities.

### 3.2. HPLC Characterization and Quantification of the Main Constituents from AEVM

**Figure 1:**
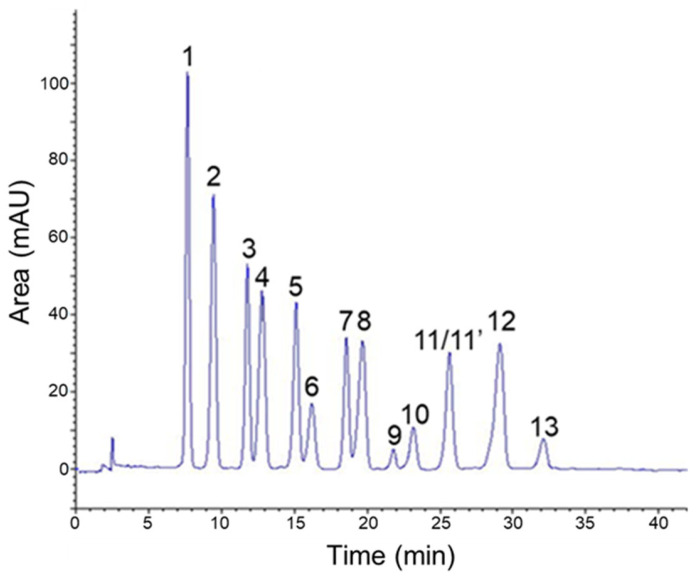
HPLC profile of anthocyanin-rich extract from *Vaccinium myrtillus* fruit with detection at 530 nm. 1: delphinidin 3-*O*-galactoside, 2: delphinidin 3-*O*-glucoside, 3: cyanidin 3-*O*-galactoside, 4: delphinidin 3-*O*-arabinoside, 5: cyanidin 3-*O*-glucoside, 6: petunidin 3-*O*-galactoside, 7: cyanidin 3-*O*-arabinoside, 8: petunidin 3-*O*-glucoside, 9: peonidin 3-*O*-galactoside, 10: petunidin 3-*O*-arabinoside, 11: peonidin 3-*O*-glucoside, 11′: malvidin 3-*O*-galactoside, 12: malvidin 3-*O*-glucoside, 13: malvidin 3-*O*-arabinoside.

Reversed-phase HPLC is a commonly employed technique for separation and analysis of complex mixtures of anthocyanins [27]. It is worth mentioning that most published protocols report the use of highly acidic mobile phases. Indeed, such a marked acidification has been shown to allow more sensitive detection as well as better separation by generating the charged and colored flavylium form of anthocyanin derivatives [28]. Consequently, mixtures of phosphoric acid, water and acetonitrile have been widely investigated to highlight the constancies in the chromatographic behavior of this class of phenolics [29]. Therefore, by using that kind of mobile phase composition and under the most favorable gradient of elution, a suitable resolution was obtained and all the major peaks of AEVM reached base-line separation (Figure 1).

Analysis of UV-spectral data as well as comparison with anthocyanin standards and previous chemical investigations led to the characterization of 14 major anthocyanidin glycosides [30,31]. Consistently with previous reports on *V. myrtillus* composition [3], a substantial diversity of anthocyanidin structures was noticed as attested by the presence of delphinidin, cyanidin, petunidin, malvidin and peonidin glycosides (Table 1). Indeed, three different 3-*O*-glycosides of delphinidin were detected, including galactoside (**1**), glucoside (**2**), and arabinoside (**4**). Analogous glycosylation patterns and retention orders were also observed for cyanidin (**3**, **5**, and **7**) and petunidin (**6**, **8**, and **10**) constituents. In addition, peonidin 3-*O*-galactoside (**9**) as well as 3-*O*-glucoside and 3-*O*-arabinoside derivatives of malvidin (**12** and **13**) were identified. As previously reported for this same extract [30,31], a co-elution occurred for peonidin 3-*O*-glucoside (**11**) and malvidin 3-*O*-galactoside (**11′**) explaining the lack of quantitative analysis for these two constituents. From a quantitative point of view, compounds **1** and **2** were shown to be the most abundant ones with respective contents of 45.18 mg/g and 45.07 mg/g of dry extract. Additionally, with global content of 125.36 mg/g, delphinidin glycosides were the most represented anthocyanin group among AEVM.

### 3.3. DPPH Spiking Experiments for Detection of Main Radical Scavengers from AEVM

**Figure 2:**
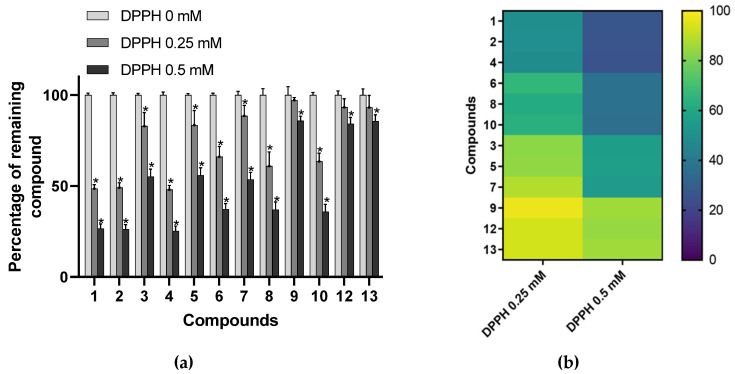
(**a**) Percentage of remaining compounds after incubation of the extract with various concentrations of DPPH (0, 0.25, and 0.5 mM). Data are presented as means ± SEM (*n* = 3). * *p* < 0.05 vs. control (DPPH = 0 mM); (**b**) Heat map representation of the percentage of remaining compounds clustered according to their aglycone moieties: delphinidin (1, 2, 4), petunidin (6, 8, 10), cyanidin (3, 5, 7), peonidin (9), and malvidin (12, 13).

DPPH spiking assay has been described as a rapid and reliable method to detect antioxidant constituents in complex biological matrices [32]. This kind of experiment is based on the fact that radical scavenging compounds are structurally modified after reaction with radical species. Consequently, the peak area of bioactive constituents is lessened in the HPLC chromatogram of DPPH pretreated extracts. Spiking experiments with a 0.25 mM concentration of radical resulted in a significant decrease (*p* < 0.05) of peak areas of compounds **1**, **2**, **3**, **4**, **5**, **6**, **7**, **8**, and **10** (Figure 2). All of these constituents corresponded to delphinidin (**1**, **2**, and **4**), cyanidin (**3**, **5**, and **7**) or petunidin (**6**, **8**, and **10**) glycosides. By contrast, peonidin (**9**) and malvidin (**12** and **13**) derivatives were not impaired by radical pretreatment at that concentration. Of interest, data showed that no significant differences were observed in the reactivity of derivatives with analogous aglycones, indicating that the nature of the sugar moiety might not influenced the antioxidant activity of bilberry anthocyanins. Besides, a second experiment performed with a higher concentration of DPPH radical (0.5 mM) led to a significant modification to the peak areas of all the compounds of the extract. This implies that every anthocyanin constituent of AEVM can be considered as potential radical scavengers. Therefore, it appears that their effects noticeably differ depending on the substitution pattern of their aglycone moieties. Indeed, as illustrated in Figure 2, data indicated that delphinidin glycosides were the most affected compounds followed by petunidin and cyanidin derivatives. By contrast, minor diminutions were observed for malvidin and peonidin glycosides, suggesting that these two groups of anthocyanins might exert lower DPPH radical scavenging activities. From a structural point of view, it is interesting to note that the degree of hydroxylation in the B ring markedly affects the reactivity of these constituents. Indeed, with three adjacent hydroxyl groups in the 3′, 4′, and 5′ positions of ring B (i.e., pyrogallol pattern), delphinidin derivatives were demonstrated to be the most reactive components of the extract. They were followed by petunidin and cyanidin glycosides, which are only hydroxylated in positions 3′ and 4′. As reported above, these catechol-type anthocyanins were also deeply affected by radical pretreatment. Conversely, compounds bearing a single free hydroxyl group in position 4′ (malvidin and peonidin glycosides) were shown to be the least reactive ones. Of interest, such observation is consistent with several previous investigations of structure-DPPH radical scavenging activity relationships investigations indicating that the radical scavenging activity of anthocyanin and flavonoid derivatives is increased with the number of hydroxyl groups on B ring [11,33,34].

### 3.4. MGO Spiking Experiments for Detection of Main Dicarbonyl Trapping Constituents from AEVM

**Figure 3:**
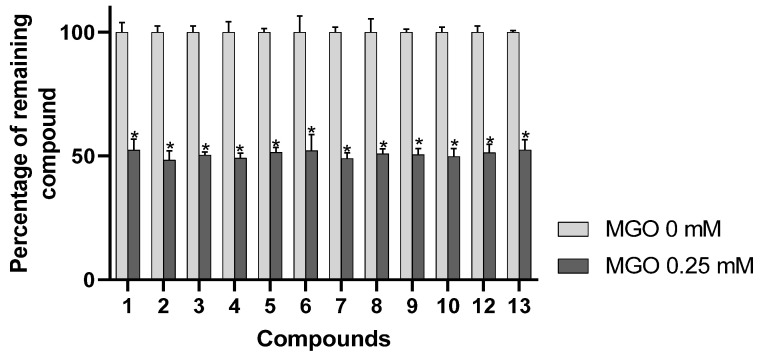
Percentage of remaining compounds after incubation of the extract with methylglyoxal (MGO, 0.25 mM). Data are presented as means ± SEM (*n* = 3). * *p* < 0.05 vs. control (MGO = 0 mM).

α-dicarbonyl compounds such as MGO are considered to act as major contributors to AGE formation in foods as well as in vivo [8,35]. Interestingly, direct trapping of these highly reactive carbonyl species has been shown to constitute an effective strategy for limiting AGEs formation [7]. Therefore, the application of a method allowing straightforward detection of MGO scavenging constituents from complex plant extracts is highly valuable in order to characterize their active principles but also to clarify further the mechanism of their antiglycative action. As indicated in Figure 3, incubation of AEVM with 0.25 mM of MGO elicited a significant decrease in the areas of the 12 evaluated compounds (*p* < 0.05), confirming the antiglycation properties of AEVM constituents. However, unlike during DPPH spiking experiments, all the anthocyanins were similarly affected by MGO pretreatment, suggesting that they presented equivalent carbonyl trapping capacities. It also tends to reveal that structural differences among these derivatives might not impact their efficiencies. Such results are consistent with a previous investigation of Blackcurrant anthocyanins, which reported that 3-*O*-rutinosides of delphinidin and cyanidin both showed significant and comparable MGO scavenging capacities [7]. Of interest, by contrast with antiradical activities, the carbonyl-trapping properties of flavonoid derivatives have been shown to not correlate with the presence of vicinal phenolic functions on B ring such as catechol or pyrogallol substitution patterns [36]. Indeed, numerous investigations have determined that meta-dihydroxyl substituted aromatic nucleus constitutes the main site of scavenging action of flavonoids. Such patterns have been shown to generate nucleophilic site between to two phenolic groups that can be attacked by the electrophilic aldehyde group of MGO [37]. In line with this, structural analyses of MGO adducts of several common flavonoid derivatives including quercetin, apigenin and genistein have confirmed that positions 6 and 8 of their A ring were the major active sites for scavenging α-dicarbonyl compounds. It has to be noticed that all bilberry anthocyanins possess the same A-ring with 5,7-dihydroxyl substitution pattern, which likely constitutes the common structural feature of their carbonyl trapping properties.

### 3.5. DPPH Radical Scavenging and Antiglycative Activities of Representative Anthocyanin Standards

To further confirm the interpretations of the performed spiking assays, 3-*O*-glucosides of delphinidin, cyanidin, petunidin, malvidin and peonidin were assessed for their antioxidant and antiglycative capacities. As indicated in Table 2, DPPH scavenging activities of these constituents were highly consistent with HPLC experiments. Indeed, with respective IC_50_ values of 10.55 ± 0.06 µM and 10.97 ± 0.12 µM, delphinidin and petunidin glucosides were confirmed to exert the most potent DPPH scavenging effects. Of interest, excellent activity was also highlighted for cyanidin glycoside as attested by its IC_50_ of 11.70 ± 0.08 µM.

In the same way as for DPPH spiking experiments, weaker effects were observed for malvidin (IC_50_ = 13.33 ± 0.16 µM) and peonidin (IC_50_ = 14.87 ± 0.28 µM) derivatives. In addition, potent antiglycation activities were also pointed out for these five anthocyanidin glycosides, as attested by their IC_50_ values being substantially lower than that of aminoguanidine chloride. It is important to note that significant differences were observed among the evaluated pure compounds. Indeed, with respective IC_50_ values of 114.2 ± 7.8, 130.5 ± 2.8, and 132.4 ± 3.7 µM, cyanidin, delphinidin and petunidin glucosides induced the highest inhibitory effects on AGE formation. Besides, malvidin and peonidin derivatives were shown to exert slightly weaker inhibitory effects on AGE formation. Interestingly, these results are consistent with a previous structure-activity relationship evaluation of flavonoid constituents showing that methylation generally exert a negative influence on their antiglycation activity [38]. Considering their above-described analogous carbonyl trapping capacities, these discrepancies suggests the existence of additional contributing mechanisms to the antiglycative action. Of interest, inhibitory effects on AGE formation positively correlated with DPPH radical scavenging activities (R^2^ = 0.7695), indicating that the antioxidant capacity of bilberry anthocyanins might also be partly responsible for their antiglycative properties. It is worth mentioning that this dual mode of action has been previously reported for several phenolic derivatives, including gallocatechin derivatives and stilbene glucosides [39,40].

## 4. Conclusions

The present study points out the efficiency of the two employed pre-column HPLC evaluations for direct detection of radical and carbonyl scavenging constituents from complex plant matrices. Besides, a DPPH spiking assay has successfully highlighted the importance of aglycone moieties in the individual antioxidant activities of bilberry anthocyanins. DPPH colorimetric evaluation of 3-*O*-glucosides of delphinidin, cyanidin, petunidin, malvidin, and peonidin further supports this observation. It indeed confirms that delphinidin, petunidin, and cyanidin derivatives exert the highest radical scavenging activities as attested by their respective IC_50_ values of 10.55 ± 0.06, 10.97 ± 0.12, and 11.70 ± 0.08 µM. In addition, the MGO-HPLC assay has provided novel information regarding the mechanism of bilberry anthocyanins inhibitory action on AGEs formation and their α-dicarbonyl scavenging capacities. Taken together, these results indicate that anthocyanin constituents, and more especially delphinidin, petunidin, and cyanidin glycosides, can be regarded as potent contributors to the antiglycative properties of *V. myrtillus* fruits owing to a dual mode of action associating radical as well as carbonyl trapping effects. Hence, this investigation tends to support the potential benefits of anthocyanin consumption on both oxidative and carbonyl stresses. However, further in vivo investigations will be required to confirm the physiological relevance of these reported activities.

## Figures and Tables

**Table 1:** Retention time and UV maximum absorption of constituents of anthocyanin-rich extract from *Vaccinium myrtillus* fruit.

Peak Number	Compound	Retention Time (min)	UV, *λ*_max_ (nm)	Content (mg/g)
1	Delphinidin 3-*O*-galactoside	7.6	277-524	45.18 ± 0.20
2	Delphinidin 3-*O*-glucoside	9.4	277-524	45.07 ± 0.31
3	Cyanidin 3-*O*-galactoside	11.7	280-517	29.69 ± 0.07
4	Delphinidin 3-*O*-arabinoside	12.7	278-524	35.11 ± 0.37
5	Cyanidin 3-*O*-glucoside	15.0	280-517	31.01 ± 0.08
6	Petunidin 3-*O*-galactoside	16.1	278-524	21.00 ± 0.09
7	Cyanidin 3-*O*-arabinoside	18.4	282-517	21.68 ± 0.51
8	Petunidin 3-*O*-glucoside	19.5	277-524	35.08 ± 0.85
9	Peonidin 3-*O*-galactoside	21.6	280-518	5.33 ± 0.10
10	Petunidin 3-*O*-arabinoside	23.0	277-525	14.92 ± 0.09
11	Peonidin 3-*O*-glucoside	25.4	277-522	N.D.
11′	Malvidin 3-*O*-galactoside			N.D.
12	Malvidin 3-*O*-glucoside	28.8	277-527	41.45 ± 0.68
13	Malvidin 3-*O*-arabinoside	31.8	277-527	9.51 ± 0.25

N.D., not determined; content values are presented as means ± SEM (*n* = 3).

**Table 2:** DPPH radical scavenging and antiglycation activities of representative anthocyanins from *Vaccinium myrtillus* fruit.

Compound	DPPH Radical Scavenging Activity (IC_50_, μM)	Antiglycation Activity (IC_50_, μM)
Delphinidin 3-*O*-glucoside	10.55 ± 0.06 ^a^	130.5 ± 2.8 ^a^
Petunidin 3-*O*-glucoside	10.97 ± 0.12 ^a^	132.4 ± 3.7 ^a,b^
Cyanidin 3-*O*-glucoside	11.70 ± 0.08 ^b^	114.2 ± 7.8 ^a^
Malvidin 3-*O*-glucoside	13.33 ± 0.16 ^c^	151.5 ± 6.0 ^b^
Peonidin 3-*O*-glucoside	14.87 ± 0.28 ^d^	193.7 ± 11.7 ^c^
Trolox	17.62 ± 0.35 ^e^	N.D.
Aminoguanidine chloride	N.D.	2387.4 ± 123.7 ^d^

Data are presented as means ± SEM (*n* = 3-4). Values in the same column sharing identical superscript are not significantly different from each other (*p* > 0.05). N.D., not determined.

## References

[1] Prokop J., Lněničková K., Cibiček N., Kosina P., Tománková V., Jourová L., Láníčková T., Skálová L., Szotáková B., Anzenbacher P. (2019). Effect of bilberry extract (*Vaccinium myrtillus* L.) on drug-metabolizing enzymes in rats. Food Chem. Toxicol..

[2] Rohloff J., Uleberg E., Nes A., Krogstad T., Nestby R., Martinussen I. (2015). Nutritional composition of bilberries (*Vaccinium myrtillus* L.) from forest fields in Norway–Effects of geographic origin, climate, fertilization and soil properties. J. Appl. Bot. Food Qual..

[3] Popović D., Đukić D., Katić V., Jović Z., Jović M., Lalić J., Golubović I., Stojanović S., Ulrih N.P., Stanković M. (2016). Antioxidant and proapoptotic effects of anthocyanins from bilberry extract in rats exposed to hepatotoxic effects of carbon tetrachloride. Life Sci..

[4] Szymanowska U., Baraniak B. (2019). Antioxidant and Potentially Anti-Inflammatory Activity of Anthocyanin Fractions from Pomace Obtained from Enzymatically Treated Raspberries. Antioxidants.

[5] Miguel M.G. (2011). Anthocyanins: Antioxidant and/or anti-inflammatory activities. J. Appl. Pharm. Sci..

[6] Harris C.S., Cuerrier A., Lamont E., Haddad P.S., Arnason J.T., Bennett S.A.L., Johns T. (2014). Investigating Wild Berries as a Dietary Approach to Reducing the Formation of Advanced Glycation Endproducts: Chemical Correlates of In Vitro Antiglycation Activity. Plant Foods Hum. Nutr..

[7] Chen X.-Y., Huang I.-M., Hwang L.S., Ho C.-T., Li S., Lo C.-Y. (2014). Anthocyanins in blackcurrant effectively prevent the formation of advanced glycation end products by trapping methylglyoxal. J. Funct. Foods.

[8] Chen L., Zhang X., Wang Q., Li W., Liu L. (2019). Effect of *Vaccinium myrtillus* Extract Supplement on Advanced Glycation End-products: A Pilot Study (P06-098-19). Curr. Dev. Nutr..

[9] Habanova M., Saraiva J.A., Haban M., Schwarzova M., Chlebo P., Predna L., Gažo J., Wyka J. (2016). Intake of bilberries (*Vaccinium myrtillus* L.) reduced risk factors for cardiovascular disease by inducing favorable changes in lipoprotein profiles. Nutr. Res..

[10] Li D., Zhang Y., Liu Y., Sun R., Xia M. (2015). Purified Anthocyanin Supplementation Reduces Dyslipidemia, Enhances Antioxidant Capacity, and Prevents Insulin Resistance in Diabetic Patients. J. Nutr..

[11] Ali H.M., Almagribi W., Al-Rashidi M.N. (2016). Antiradical and reductant activities of anthocyanidins and anthocyanins, structure–activity relationship and synthesis. Food Chem..

[12] Gardana C., Ciappellano S., Marinoni L., Fachechi C., Simonetti P. (2014). Bilberry adulteration: Identification and chemical profiling of anthocyanins by different analytical methods. J. Agric. Food Chem..

[13] Yao H., Chen Y., Shi P., Hu J., Li S., Huang L., Lin J., Lin X. (2012). Screening and quantitative analysis of antioxidants in the fruits of *Livistona chinensis* R. Br using HPLC-DAD–ESI/MS coupled with pre-column DPPH assay. Food Chem..

[14] Shui G., Peng L.L. (2004). An improved method for the analysis of major antioxidants of *Hibiscus esculentus* Linn. J. Chromatogr. A.

[15] Fraisse D., Degerine-Roussel A., Bred A., Ndoye S., Vivier M., Felgines C., Senejoux F. (2018). A Novel HPLC Method for Direct Detection of Nitric Oxide Scavengers from Complex Plant Matrices and Its Application to *Aloysia triphylla* Leaves. Molecules.

[16] Könczöl Á., Kéry Á., Keserű G.M., Balogh G.T. (2010). LC Determination of Peroxynitrite Scavenging Activity of Phenols from *Salvia* spp.. Chroma.

[17] Tang D., Zhu J.-X., Wu A.-G., Xu Y.-H., Duan T.-T., Zheng Z.-G., Wang R.-S., Li D., Zhu Q. (2013). Pre-column incubation followed by fast liquid chromatography analysis for rapid screening of natural methylglyoxal scavengers directly from herbal medicines: Case study of *Polygonum cuspidatum*. J. Chromatogr. A.

[18] Nowotny K., Jung T., Höhn A., Weber D., Grune T. (2015). Advanced Glycation End Products and Oxidative Stress in Type 2 Diabetes Mellitus. Biomolecules.

[19] (2016). Bilberry Fresh Fruit. European Pharmacopoeia.

[20] Ndoye S., Fraisse D., Akendengué B., Dioum M., Gueye R., Sall C., Seck I., Felgines C., Seck M., Senejoux F. (2018). Antioxidant and antiglycation properties of two mango (*Mangifera indica* L.) cultivars from Senegal. Asian Pac. J. Trop. Biomed..

[21] Séro L., Sanguinet L., Blanchard P., Dang B., Morel S., Richomme P., Séraphin D., Derbré S. (2013). Tuning a 96-Well Microtiter Plate Fluorescence-Based Assay to Identify AGE Inhibitors in Crude Plant Extracts. Molecules.

[22] Meda N.R., Fraisse D., Gnoula C., Vivier M., Felgines C., Senejoux F. (2017). Characterization of antioxidants from *Detarium microcarpum* Guill. et Perr. leaves using HPLC-DAD coupled with pre-column DPPH assay. Eur. Food Res. Technol..

[23] Shi J., Mazza G., Le Maguer M., Functional Foods--Biochemical & Processing Aspects (1998). Functional Foods & Nutraceuticals Series.

[24] Brasanac-Vukanovic S., Mutic J., Stankovic D., Arsic I., Blagojevic N., Vukasinovic-Pesic V., Tadic V. (2018). Wild Bilberry (*Vaccinium myrtillus* L., Ericaceae) from Montenegro as a Source of Antioxidants for Use in the Production of Nutraceuticals. Molecules.

[25] Spínola V., Pinto J., Castilho P.C. (2018). Hypoglycemic, anti-glycation and antioxidant in vitro properties of two *Vaccinium* species from Macaronesia: A relation to their phenolic composition. J. Funct. Foods.

[26] McIntyre K.L., Harris C.S., Saleem A., Beaulieu L.-P., Ta C.A., Haddad P.S., Arnason J.T. (2009). Seasonal phytochemical variation of anti-glycation principles in lowbush blueberry (*Vaccinium angustifolium*). Planta Med..

[27] da Costa C.T., Horton D., Margolis S.A. (2000). Analysis of anthocyanins in foods by liquid chromatography, liquid chromatography-mass spectrometry and capillary electrophoresis. J. Chromatogr. A.

[28] Deineka V.I., Deineka L.A., Saenko I.I. (2015). Regularities of Anthocyanins Retention in RP HPLC for “Water–Acetonitrile–Phosphoric Acid” Mobile Phases. J. Anal. Methods Chem..

[29] Deineka V.I., Grigor’ev A.M. (2004). Determination of Anthocyanins by High-Performance Liquid Chromatography: Regularities of Retention. J. Anal. Methods Chem..

[30] Mauray A., Felgines C., Morand C., Mazur A., Scalbert A., Milenkovic D. (2010). Nutrigenomic analysis of the protective effects of bilberry anthocyanin-rich extract in apo E-deficient mice. Genes Nutr..

[31] Mauray A., Felgines C., Morand C., Mazur A., Scalbert A., Milenkovic D. (2012). Bilberry anthocyanin-rich extract alters expression of genes related to atherosclerosis development in aorta of apo E-deficient mice. Nutr. Metab. Cardiovasc. Dis..

[32] Zhang Y.-P., Shi S.-Y., Xiong X., Chen X.-Q., Peng M.-J. (2012). Comparative evaluation of three methods based on high-performance liquid chromatography analysis combined with a 2,2′-diphenyl-1-picrylhydrazyl assay for the rapid screening of antioxidants from *Pueraria lobata* flowers. Anal. Bioanal. Chem..

[33] Amic D., Davidovic-Amic D., Beslo D., Rastija V., Lucic B., Trinajstic N. (2007). SAR and QSAR of the Antioxidant Activity of Flavonoids. Curr. Med. Chem..

[34] Blando F., Calabriso N., Berland H., Maiorano G., Gerardi C., Carluccio M., Andersen Ø. (2018). Radical Scavenging and Anti-Inflammatory Activities of Representative Anthocyanin Groupings from Pigment-Rich Fruits and Vegetables. IJMS.

[35] Wang S. (2019). Chemical Hazards in Thermally-Processed Foods.

[36] Shao X., Chen H., Zhu Y., Sedighi R., Ho C.-T., Sang S. (2014). Essential Structural Requirements and Additive Effects for Flavonoids to Scavenge Methylglyoxal. J. Agric. Food Chem..

[37] Hwang S., Kim H., Zuo G., Wang Z., Lee J.-Y., Lim S. (2018). Anti-glycation, Carbonyl Trapping and Anti-inflammatory Activities of Chrysin Derivatives. Molecules.

[38] Xie Y., Chen X. (2013). Structures Required of Polyphenols for Inhibiting Advanced Glycation end Products Formation. Curr. Drug Metab..

[39] Ho C.-T., Wang M. (2013). Dietary Phenolics as Reactive Carbonyl Scavengers: Potential Impact on Human Health and Mechanism of Action. J. Tradit. Complement Med..

[40] Liu M., Tang F., Cao H., Xiao J., Chen X. (2017). Inhibition of Resveratrol Glucosides against AGEs Formation. Free Radical Bio. Med..

